# Editorial: Reviews in rheumatology

**DOI:** 10.3389/fmed.2023.1153419

**Published:** 2023-02-21

**Authors:** Yves Henrotin, Zoltan Szekanecz, Kayo Masuko

**Affiliations:** ^1^musculoSKeletal Innovative Research Lab (mSKIL), University of Liège, Liège, Belgium; ^2^Artialis SA, Liège, Belgium; ^3^Princess Paola Hospital, Department of Physical Therapy and Rehabilitation, Vivalia, Marche-en-Famenne, Belgium; ^4^The Osteoarthritis Foundation, Boncelles, Belgium; ^5^Department of Rheumatology, Faculty of Medicine, University of Debrecen, Debrecen, Hungary; ^6^Akasaka Sanno Medical Center, Tokyo, Japan

**Keywords:** rheumatoid arthritis, systemic lupus erythematosus (SLE), frozen shoulder, cytokines, biomarkers, mitochondria

Rheumatic diseases and musculoskeletal disorders are among the leading causes of disability worldwide. It is therefore essential to promote medical and scientific advances in these diseases (1). This is why we initiated the special edition research series in Frontiers in Medicine called “*Review in rheumatology*.” This series aims to overview the most recent advances in the physiopathology, diagnosis, and treatment of rheumatic diseases and musculoskeletal disorders. The authors were also invited to propose future directions for basic and clinical research. Four published papers were dedicated to rheumatoid arthritis, one to systemic lupus erythematosus, and finally one on frozen shoulder. These pathologies have several pathophysiological mechanisms in common, such as the activation of the innate immune response, and local and systemic inflammation. Control inflammation is a key target in the management of these pathologies not only to control symptoms but also to prevent connective tissue degradation. The knowledge of inflammation has strongly progressed during the last decades. Macrophage polarization, mitochondria homeostasis, intestinal or oral dysbiosis, cell senescence, and environmental factors are among the most investigated possible therapeutic target to resolve inflammation chronicity and therapeutic failure in patients with severe rheumatic diseases like rheumatoid arthritis (RA).

Difficult-to-treat RA (D2T RA) is a specific disease state that affects 3%−10% of RA patients. This disease state can be defined as the persistence of signs suggestive of active/progressive disease after conventional synthetic disease-modifying antirheumatic drug therapy (csDMARDs) and at least two targeted biologic and synthetic DMARDs (b/ts) with different mechanisms of action. Watanabe et al. discussed this particular unresolved disease state and proposed a strategy to prevent patients from developing this D2T RA. According to them, the treatment of D2T RA requires a combination of pharmacological and non-pharmacological interventions. Correcting modifiable risk factors, such as smoking, obesity, poor compliance, and periodontitis, is also important in suppressing the development of RA D2T. Starting early intervention with b/tsDMARDs and selecting the appropriate b/tsDMARDs are two other essential actions to prevent the onset of this particular disease condition, especially for patients with highly active disease who have an inadequate response to high-dose of methotrexate.

Osteoimmunology concepts in RA account for the molecular crosstalk between the immune and skeletal systems, resulting in the disruption of bone remodeling. In their review, Sakthiswary et al. summarized the molecular mechanisms involved in bone loss associated with RA with particular attention to the aryl-hydrocarbon receptor (Ahr) which acts on this process through the generation of Th17 and the RANKL/OPG balance. A higher RANKL/OPG ratio is associated with increased radiographic damage in RA patients, suggesting that controlling this balance may prevent bone resorption in RA.

Interleukin-34 (IL-34) is a macrophage growth factor detected at high levels in synovial fluid (SF), synovial cells, and serum of RA patients. The role of this cytokine in controlling the inflammatory response remains controversial. For some authors, it is pro-inflammatory and for others anti-inflammatory. IL-34 is also proposed as a biomarker of RA remission due to a strong correlation between IL-34 and RA relapse after discontinuation of antirheumatic therapy. In this article, Park et al. amalgamate information surrounding the involvement of IL-34 in the pathogenesis of RA, in particular regarding how IL-34 integrates into the mechanisms of macrophage and T-cell activation seen in RA.

Modulation of mitochondrial homeostasis and repair of mitochondrial damage to treat RA is a very promising therapeutic approach. In their article, Cui et al. address several research ways such as the modulation of biogenesis, function, and autophagy of mitochondria. To date, most frontier anti-rheumatic drugs commonly used clinically, like methotrexate, maintain mitochondrial homeostasis. In contrast, many targeted biological therapies for RA cannot directly target mitochondria. Rather, they block cytokine signaling pathways, especially inflammatory cytokine signaling, which has a powerful effect on mitochondrial biology. Therefore, molecules targeting specifically mitochondria could open up new therapeutic avenues.

Systemic lupus erythematosus (SLE) is a disease associated with a high risk of mortality mainly due to lupus nephritis. Diagnostic delays and misdiagnoses are common in SLE. Early diagnosis and effective treatment may prevent long-term complications that cause increased morbidity and mortality. Yavuz and Lipsky discussed the discovery and the relevance of clinical and molecular markers for early diagnosis, monitoring of disease, and phenotyping of the SLE patient to deliver the appropriate therapy based on specific dysregulated molecular pathways.

Finally, de la Serna et al. proposed an intriguing paper about the physiopathology of a frozen shoulder. They hypothesize that idiopathic frozen shoulder and comorbid conditions such as diabetes share common pathophysiological mechanisms, such as the accumulation of advanced glycation end products derived from insulin resistance and low-grade inflammation. This hypothesis opens new therapeutic perspectives. Besides the core treatment of the freezing phase combining corticosteroids infiltration, manual mobilization, and physiotherapy, the authors suggest the administration of lactoferrin, pre-probiotics, glutamine supplementation, curcumin, resveratrol, EGCG, soy isoflavones, etc., as potential modulators of the disease, although further studies are warranted.

In conclusion, the articles published in this Research Topic report the most recent knowledge on the pathophysiology of some of the most debilitating inflammatory rheumatic diseases. They identify exciting future directions for their diagnosis and treatment. We sincerely hope that readers will enjoy their reading and that it will be inspiring for their clinical and research activity.

## Author contributions

All authors listed have made a substantial, direct, and intellectual contribution to the work and approved it for publication.
