# Corrigendum: Editorial: Reviews in rheumatology

**DOI:** 10.3389/fmed.2023.1187603

**Published:** 2023-03-29

**Authors:** Yves Henrotin, Zoltan Szekanecz, Kayo Masuko

**Affiliations:** ^1^musculoSKeletal Innovative Research Lab (mSKIL), University of Liège, Liège, Belgium; ^2^Artialis SA, Liège, Belgium; ^3^Princess Paola Hospital, Department of Physical Therapy and Rehabilitation, Vivalia, Marche-en-Famenne, Belgium; ^4^The Osteoarthritis Foundation, Boncelles, Belgium; ^5^Department of Rheumatology, Faculty of Medicine, University of Debrecen, Debrecen, Hungary; ^6^Akasaka Sanno Medical Center, Tokyo, Japan

**Keywords:** rheumatoid arthritis, systemic lupus erythematosus (SLE), frozen shoulder, cytokines, biomarkers, mitochondria

In the published article, there was an error in affiliations 4, 5 and 6.

Instead of “^5^Akasaka Sanno Medical Center, Tokyo, Japan, ^6^Rheumatology Department, University of Debrecen, Debrecen, Hungary.”

It should be “^5^Department of Rheumatology, Faculty of Medicine, University of Debrecen, Debrecen, Hungary, ^6^Akasaka Sanno Medical Center, Tokyo, Japan.”

And, Instead of “Szekanecz^4,5^, Masuko^5,6^,” it should be “Szekanecz^5^, Masuko^6^.”

In the published article, there was an error in the Conflict of Interest statement. “YH and ZS were employed by the Osteoarthritis Foundation.” The correct Conflict of Interest statement appears below.

The authors apologize for this error and state that this does not change the scientific conclusions of the article in any way. The original article has been updated.

